# Pulsed Laser Photo-Crosslinking of Gelatin Methacryloyl Hydrogels for the Controlled Delivery of Chlorpromazine to Combat Antimicrobial Resistance

**DOI:** 10.3390/pharmaceutics14102121

**Published:** 2022-10-06

**Authors:** Tatiana Tozar, Simona Nistorescu, Mihai Boni, Gratiela Gradisteanu Pircalabioru, Irina Negut, Angela Staicu

**Affiliations:** 1 Laser Department, National Institute for Laser, Plasma and Radiation Physics, 409 Atomistilor, 077125 Magurele, Romania; 2 Department of Biochemistry and Molecular Biology, Faculty of Biology, University of Bucharest, 91–95 Splaiul Independentei, 050095 Bucharest, Romania; 3 Research Institute of the University of Bucharest (ICUB), University of Bucharest, 050095 Bucharest, Romania; 4 Academy of Romanian Scientists, Ilfov Street 3, 050054 Bucharest, Romania

**Keywords:** Irgacure 2959, 266 nm pulsed laser exposure, GelMa, hydrogel, photopolymerization, antimicrobial effect, cytotoxicity

## Abstract

Hydrogels are ideal candidates for the sustained local administration of antimicrobial drugs because they have customizable physicochemical properties that allow drug release kinetics to be controlled and potentially address the issue of systemic side effects. Consequently, the purpose of this study was to use 266 nm-pulsed laser beams to photo-crosslink gelatin methacryloyl hydrogels using Irgacure 2959 as a photo-initiator to reduce the curing time and to have an online method to monitor the process, such as laser-induced fluorescence. Additionally, irradiated chlorpromazine was loaded into the hydrogels to obtain a drug delivery system with antimicrobial activity. These hydrogels were investigated by UV–Vis and FTIR absorption spectroscopy, scanning electron microscopy, and laser-induced fluorescence spectroscopy and their structural and morphological characteristics, swelling behavior, and drug release profile were obtained. As a result the morphology, swelling behavior, and drug release profile were influenced by both the energy of the laser beam and the exposure time. The optimal hydrogel was obtained after 1 min of laser irradiation for Irgacure 2959 at 0.05% *w*/*v* concentration and gelatin methacryloyl at 10% *w*/*v* concentration. The hydrogels loaded with irradiated chlorpromazine show significant antimicrobial activity against *Staphylococcus aureus* and MRSA bacteria and a non-cytotoxic effect against L929 fibroblast cell lines.

## 1. Introduction

Traditional drug therapy frequently requests high doses or repeated administration to produce a therapeutic effect; this can reduce the overall efficacy and patient compliance, as well as cause significant adverse effects and even toxicity [1,2]. In one clinical trial, intravenously injected interleukin-12 caused systemic toxicity and two patients had died [1]. On the other hand, oral administration, which is the most commonly used method of medication delivery, has significant limitations, including poor targeting and short circulation times (less than 12 h) [2].

Controlled drug delivery methods, such as membranes, micelles, nanoparticles, liposomes, and hydrogels, have been the focus of recent research to overcome these challenges; they can improve therapeutic efficacy and also reduce toxicity and dosage requirements [3]. Gellan gum polysaccharide functionalized with sericin and rice bran albumin protein was used as polysaccharides-protein nanocomposite for controlled delivery of doxorubicin to kill MCF-7 breast cancer cells. As a result, doxorubicin-loaded nanocarriers had a greater cytotoxic effect than free doxorubicin [4]. Another approach involves the fabrication of ligand-coupled micelles into biodegradable nanofibers for targeted cancer drug delivery. In this respect, poly(bis(carbox-yphenoxy)phosphazene-cholic acid polymer micelles loaded with paclitaxel were incorporated in psyllium husk mucilage nanofibers. The paclitaxel released inhibited the growth of MCF-7 breast cancer cells by the generation of reactive oxygen species and by cell cycle arrest [5]. Similar to this, gelatin-based drug delivery systems could be created for therapeutic drugs’ targeted and controlled delivery. Gelatin modified with furfuryl amine together with eosin Y and/or riboflavin were subjected to an LED emitting at 535 nm and due to photo-oxidation reaction were converted into hydrogels for controlled release of ceftriaxone. The results showed that when Eosin-Y was used as a crosslinker, an excellent, smart hydrogel system was created, having improved encapsulation efficiency [6]. In the same manner, photo-crosslinked gelatin methacryloyl (GelMa) hydrogels were used as injectable delivery systems for chlorhexidine in regenerative endodontic therapy. The findings demonstrated significant antimicrobial action of the chlorhexidine loaded GelMa hydrogels against *Actinomyces naeslundii* ATCC 12104 and *Enterococcus faecalis* ATCC 19433, having the potential to be used as a biocompatible injectable drug delivery system for root canal disinfection [7]. 

One of the applications of controlled drug delivery systems is represented by the topical application. Compared to oral and injectable drug delivery, topical drug delivery systems have various benefits such as preventing changes of the drug in plasma concentrations, gastric pH alterations, or first-pass metabolism in the liver [8,9,10]. A topical drug delivery system is intended to treat local diseases by administering medicines to superficial body areas such as the skin, eyes, and nose [10]. Millions of people in the USA experience skin diseases every day, such as bacterial, fungal, viral, and autoimmune conditions [11,12]. Skin conditions cost more and are more common than other illnesses that pose serious public health risks, such as diabetes and cardiovascular disease [11]. The local therapy of skin disorders involves ongoing research on topical medicine delivery to the skin’s surface. In this regard, various approaches for drug delivery are proposed, such as gels or creams [13], hydrogels [14], nano-colloidal carriers [15], and microneedles [16]. 

Hydrogels are three-dimensional polymeric networks that can be used as drug delivery systems in a variety of medical fields such as cardiology, cancer, immunology, wound healing, and pain relief [17,18]. The high-water content of hydrogels (typically 70–90%) offers biocompatibility, physical similarity to the tissue, and encapsulation of hydrophilic medicines. When water is used as a solvent, the risk of aggregation and drug denaturation that occurs in organic solvents is avoided [19]. The UV-curing method for hydrogel synthesis is widely used in a variety of industries due to its benefits such as reduced pollution, lower energy consumption, and faster curing. To obtain hydrogels using this method, a mixture of natural or synthetic polymer and an initiator is used [20]. Photopolymerization uses light to dissociate the initiator molecules into free radicals, which react with the double bonds of the polymers, causing crosslinking [21]. Furthermore, to enhance the photo-crosslinking process, most polymers are chemically altered, typically by inserting functional groups, as in the widely used modified poly(ethylene)glycol diacrylates [22]. As for the photoinitiators, even at low percentage loading, they have a significant impact on curing speed and the quality of the hydrogel. Briefly, photoinitiator molecules break into free radicals when exposed to visible or UV light at specific wavelengths, which starts the polymerization reaction. The photoinitiator is excited or dissociated into a high-energy radical state by a photon from a light source. This radical then causes a macromer solution to polymerize. However, the generation of a high-energy radical species in this system has the potential to cause oxidative damage in the case of photo-encapsulated cells [23].

Chemical modification of conventional radical photoinitiators, which consists of adding suitable groups to the structure of the photoinitiator, is one of the most fundamental ways to improve their solubility. The hydroxyl group is the most frequent solubilizing group, and it is found in the most popular water-soluble initiator, Irgacure 2959 (2-hydroxy-1-[4-(2-hydroxyethoxy) phenyl]-2-methyl-1-propanone). This photoinitiator has ketone groups as functional groups and is one of the first commercially available water-soluble photoinitiators. Despite its disadvantages, such as low water solubility of up to 2% and a limited absorption range (UVA up to 365 nm), this initiator has become widely used because of its cytocompatibility properties with various cell types [24,25]. 

Irgacure 2959 is a type I photoinitiator that, when exposed to light, breaks down into two radicals: benzoyl and alkyl, both of which can start a polymerization process [26]. Irgacure 2959 is commonly used as a photoinitiator for hydrogel made from poly(ethylene glycol) diacrylate, GelMa, and methacrylated hyaluronic acid [27,28]. This initiator is also used to encapsulate cells, transport medicines and cells to specific locations, and make scaffolds for cell cultures [29,30,31]. 

GelMa has become an appealing option to mimic the extracellular matrix when it is primarily made of collagen [32]. Collagen is hydrolyzed to produce gelatin that may retain relevant moieties such as arginyl-glycyl-aspartic acid, which are crucial sites for cell attachment. Further, methacrylate and/or methacrylimide groups can be added to create versatile materials that resemble the cellular matrix [33]. Regarding the photo-crosslinking of GelMa, there are many different conditions reported in the literature. The most widely used photoinitiator is Irgacure 2959 [24,25], though other ones have been used as well, such as 2,20-azobis [2-methyl-*N*-(2hydroxylethyl)propianamide] [34], lithium phenyl-2,4,6-trimethylbenzoylphosphinate [7,35], eosin Y [6], or riboflavin [6].

In this context, this paper presents original results on the effect of 266 nm pulsed laser beams on the photo-crosslinking of GelMa hydrogels when Irgacure 2959 is used as a photoinitiator and the ability of unirradiated/irradiated chlorpromazine (CPZ) loaded hydrogels to inhibit the growth of *Staphylococcus aureus* (*S. aureus*) wild type and methicillin-resistant strains (MRSA).

When using Irgacure 2959, hydrogels are typically produced using a 365 nm UV lamp. The exposure time to UV radiation depends on the type and concentration of polymer used, the concentration of Irgacure 2959, the volume of solution to be polymerized, and the light irradiance. In the case of GelMa, where its concentration ranged from 2.5% to 20% (*w*/*v*) and that of Irgacure from 0.05% to 1% (*w*/*v*), the exposure time was between 4–290 s for an irradiance of 2–150 mW/cm^2^ [33]. Other studies revealed that for a 20% concentration of GelMa and a 0.05% Irgacure 2959 the exposure time was of 30 min at irradiance of 2.6 mW/cm^2^ and mold volume of 35 µL [36] and 5 min at 3.5 mW/cm^2^ and a mold volume of 50 µL [37].

In this study we photo-crosslinked GelMa hydrogel by exposing solutions of Irgacure (0.05%)-GelMa (10%) (*w*/*v*) to 266 nm pulsed lasers at energies of 0.25, 0.45, 0.75, and 1 mJ for time intervals between 1 and 30 min. By using lasers, the photo-crosslinking process can be significatively shortened. In addition, when pulsed lasers are used, real-time laser-induced fluorescence monitoring can help in the estimation of the rate of hydrogel formation via the rate of degradation. The rate of degradation was determined from the Irgacure fluorescence intensity and fluorescence wavelength. The resulting GelMa hydrogels were investigated in terms of swelling, permeability, and drug release. The best conditions for obtaining GelMa hydrogels were found to be 0.75 mJ laser beam energy and 1 min exposure time. Further, the GelMa hydrogels and the mixture polymer-photoinitiator were investigated by UV–Vis absorption spectroscopy, FTIR absorption spectroscopy, scanning electron microscopy (SEM), and laser-induced fluorescence (LIF) spectroscopy.

The end goal of this study was to assess the potential of the obtained hydrogels as drug delivery systems for skin-infected wounds. In this respect, hydrogels were loaded with chlorpromazine (CPZ) and CPZ exposed to 266 nm laser radiation. The choice of this drug is based on the authors’ previous studies where irradiated CPZ proved to be effective in killing drug-resistant bacteria. The use of 266 nm laser beams to irradiate CPZ solutions to generate antimicrobial photoproducts demonstrates the promising potential of this method for developing antimicrobial agents to combat drug-resistant pathogens [38,39,40,41,42]. To mimic a wound infection, the loaded hydrogels were tested against *S. aureus* ATCC 25923 and *S. aureus* MRSA, known to be responsible for the most frequent skin infections [43,44].

## 2. Materials and Methods

### 2.1. Materials

Irgacure 2959 (2-hydroxy-1-[4-(2-hydroxyethoxy) phenyl]-2-methyl-1-propanone), henceforth referred to in this study as Irgacure, was obtained from Sigma-Aldrich (St. Louis, MO, USA) and the solvent used was ultrapure water. Gelatin methacryloyl (GelMA), gel strength 300 g bloom and 80% degree of substitution, was obtained from Sigma-Aldrich, and the solvent used was ultrapure water.

Chlorpromazine (CPZ) has a purity ≥98% and was acquired in hydrochloride form also from Sigma-Aldrich. CPZ was irradiated prior to its loading into the hydrogels. A 2 mL volume of CPZ solutions was irradiated in a quartz cuvette (optical path 1 cm) under continuous stirring (700 rpm) for 30 min with a 266 nm pulsed beam emitted by Nd:YAG laser (10 Hz repetition rate, 6 ns full time width at half maximum) following the irradiation protocol described by Alexandru et al. [38]. The energy of the pulsed laser beam was 6.5 mJ and the irradiance was 171 mW/cm^2^.

Detailed analysis of the photochemical degradation of CPZ irradiated with 266 nm laser beam at 6.5 mJ beam energy and the characterization of the photoproduct mixture are presented in Alexandru et al. [38]. In brief, seven photoproducts were identified: CPZ sulfoxide, promazine, 2-hydroxy promazine, promazine sulfoxide, 2-hydroxy promazine sulfoxide, and two compounds with *m*/*z* values of 292 and 308 amu. For the 30 min irradiated CPZ the relative concentration of each product was 21.9% for CPZ, 43.2% for promazine, 14.7% for 2-hydroxy promazine/promazine sulfoxide, 6.1% for promazine sulfoxide, 4.8% CPZ sulfoxide, and 2.2% for 292 and 308 amu compounds.

The hydrogels were immersed for 24 h in 1 mL solutions of unirradiated and irradiated CPZ at concentration of 2 mg/mL.

Penicillin G sodium 1,000,000 UI obtained from Antibiotice (Antibiotice Iasi, Iasi, Romania) was used at a concentration of 2 mg/mL and loaded into the hydrogels as reference for comparison its antimicrobial activity with that of unirradiated and irradiated CPZ-loaded hydrogels.

Water was preferred as a solvent because it is safe and affordable, and it delivers a reaction efficiency that conventional organic systems cannot match in many circumstances.

### 2.2. Hydrogel Photo-Crosslinking

GelMa-Irgacure solutions were mixed with a magnetic stirrer at 200 rpm for 30 min at a temperature of 70 °C to allow the correct dissolution of each component. The concentrations tested for Irgacure were 0.05%, 0.35% and 0.7% *w*/*v* and for GelMa 10% *w*/*v*. A volume of 35 µL of the solution was placed in a mold and exposed to pulsed laser radiation emitted at 266 nm. The mold was 3D-printed from polylactic acid and had an inner diameter of 0.7 cm and a height of 0.1 cm.

The irradiation source was the fourth harmonic of a Nd:YAG laser from Continuum (San Jose, CA, USA) (10 Hz, 6 ns FWHM) emitting at 266 nm. Irgacure and Irgacure-GelMa solutions were exposed to 266 nm for 1, 5, 10, 20 and 30 min at beam energies of 0.25 mJ (irradiance, I = 6.6 mW/cm^2^), 0.45 mJ (I = 11 mW/cm^2^), 0.75 mJ (I = 19.7 mW/cm^2^) and 1 mJ (I = 26.3 mW/cm^2^).

**Figure 1:**
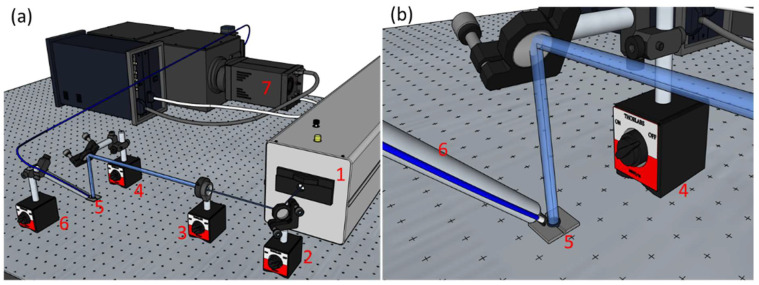
(**a**) The irradiation setup used to photo-crosslink hydrogels with 266 nm laser beams and the LIF signal collection of the fluorescence emission of the initiator. (**b**) closeup of the LIF signal collection. Legend: (1) Nd:YAG laser, (2) dielectric mirror, (3) lens and diaphragm, (4) dielectric mirror, (5) mold, (6) optical fiber, (7) spectrograph.

Figure 1 depicts the experimental setup for the irradiation. Two dielectric mirrors (NB1-K04, Thorlabs, Newton, NJ, USA) were used to direct the laser beam, a lens (LF4370, Thorlabs, focal distance, F = −150.0 mm) was used to expand the beam, and a diaphragm was used to achieve a 0.7 cm beam diameter.

The LIF signal was collected using an optical fiber (M93L02, Thorlabs, core diameter 1500 μm) positioned at 45° to the incident beam and recorded using a spectrograph SpectraPro SP-2750 (Acton Research, Czerny–Turner configuration, Trenton, NJ, USA) coupled with an ICCD camera PIMAX 1024RB (Princeton Instruments, Trenton, NJ, USA). The fluorescence spectrum was averaged over 50 recorded signals.

### 2.3. Hydrogel Characterization

Hydrogels were removed from the mold and immersed in ultrapure water (3 mL) for 24 h at room temperature to remove precursor residues and to achieve absorption equilibrium. After hydrogel extraction, the remaining solutions were analyzed for the detection of unreacted compounds. The hydrogels were dried in a desiccator for 24 h and then stored in the desiccator at 4 °C in the dark. The amount of non-reactivated Irgacure after photopolymerization was determined by UV–Vis absorption spectroscopy. The dried hydrogels were investigated by FTIR absorptions’ spectroscopy and scanning electron microscopy.

A Perkin Elmer (PerkinElmer, Inc., Waltham, MA, USA) spectrophotometer, Lambda 950 model, was used to record absorption spectra between 200 and 400 nm with a resolution of 1 nm. The optical path of the spectrophotometric cell used was 1 mm. To avoid obtaining a saturated signal for 0.7% concentrations, the spectra were recorded at 0.05% dilutions.

A Nicolet iS50 FTIR spectrometer (Thermo Fisher Scientific, Waltham, MA, USA) was used to record the IR spectra of the Irgacure solutions, dried hydrogels, and powder forms of Irgacure and GelMa. The Irgacure solutions IR spectra were measured in transmittance mode in the spectral range 1800–600 cm^−1^, with a resolution of 4 cm^−1^ and an average of 32 spectra. A volume of 20 µL of the liquid sample was applied on a KRS-5 support and dried in atmospheric conditions. The KRS-5 spectrum was subtracted from the final spectrum. The dried hydrogels and powder form samples spectra were recorded in attenuated total refraction mode in the spectral range 1800–700 cm^−1^, with a resolution of 4 cm^−1^ and an average of 16 spectra. A ZnSe crystal having 1.5 mm diameter, depth of penetration 2.03µm at 1000 cm^−1^, 2.4 refractive index, and one internal reflection at a 42° incidence angle was used.

The surface morphology of the hydrogels was inspected by a FEI Inspect S electron microscope (FEI Company, Hillsboro, OR, USA). The investigations were conducted in top-view mode at 20 kV acceleration voltages in a high-vacuum environment using secondary electron mode. To reduce electrical charging during analysis, a thin gold film was applied to the top of each hydrogel.

The swelling behavior of each hydrogel was investigated. Swelling is defined as the amount of water taken up by the hydrogel and is an indicator of the hydrophilicity of the polymer network and the relative density of crosslinking, where stiffer networks usually have lower swelling rates. According to the Japanese industry standard K8150, the dry hydrogel is immersed in deionized water for 48 h at room temperature and the swelling is calculated as follows [45]:

(1) $$\mathrm{swelling}\;=\frac{M_{\mathrm{wet}}-M_{\mathrm{dry}}\;}{M_{\mathrm{dry}}}$$

where M_wet_ = weight of the hydrogel in the swollen state and M_dry_ = weight of the hydrogel in the dry state.

The dried hydrogels were incubated in one mL of 2 mg/mL unirradiated and irradiated CPZ for 24 h at room temperature. The hydrogels were then removed, placed on a vessel, dehydrated, and stored in a desiccator at 4 °C in the dark.

Phosphate-buffered saline (PBS) is used to elucidate how the osmotic pressure varies when the hydrogels come into contact with tissues in vivo [46].The CPZ-loaded hydrogels were immersed in 1 mL PBS solution and incubated at 37 °C for 2, 4, 8, 24, and 48 h. After each time interval, the solutions were measured using UV–Vis absorption spectroscopy. The amount of drug released represents the drug concentration in the sample multiplied by the volume of the solution in which the sample was eluted [47].

### 2.4. Theoretical Absorption Spectra of Photoinitiator Simulations

The optimized molecular structures were obtained using the electronic structure calculation software Gaussian09 [48]. For absorption spectra simulations, Density Functional Theory (DFT) was applied along with B3LYP functional and B-311G(d,p) basis set using the molecular structure IEFPCM (Integral Equation Formalism Polarizable Continuum Model) model for solvation effects of water [49].

### 2.5. In Vitro Biological Evaluation

#### 2.5.1. Antimicrobial Assays

The antimicrobial activity of unirradiated and irradiated CPZ-loaded hydrogels was studied on standard and clinical isolates of gram-positive bacteria *Staphylococcus aureus* (*S. aureus*) ATCC 25923 and methicillin-resistant *S. aureus* (MRSA).

In diagnostic labs, the disk diffusion method is used to determine whether bacteria isolated from a patient’s infection are susceptible to clinically approved antibiotics [50,51]. The test was performed by inoculating the surface of an agar plate with *S. aureus* suspension. Following the application of the hydrogels to the agar, the plate was incubated for 18 h at 37 °C. The susceptibility of the bacterial isolates to each hydrogel was then quantified by measuring the area of the inhibition zones using ImageJ software (version 1.53, National Institutes of Health, Bethesda, MD, USA).

Further, the bacterial adherence to the hydrogels was determined through the viable cell counts method [52,53]. The bacterial suspensions, 10^6^ CFU/mL, were prepared in PBS. The control hydrogel, unirradiated and irradiated CPZ-loaded hydrogels were incubated 24 h and 48 h at 37 °C together with 1 mL bacterial suspension. Following that, the hydrogels with attached bacteria were extracted, immersed in 1 mL PBS, and vortexed. Serial dilutions of the samples were plated onto a Plate Count Agar and incubated overnight at 37 °C. By counting the number of colonies from the plate, the quantity of bacteria in the initial sample was calculated taking into account also the dilution factor. For comparison purposes, hydrogels loaded with penicillin were analyzed and their antimicrobial activity was compared with CPZ unirradiated/irradiated loaded hydrogels.

#### 2.5.2. Cell Viability and Morphology Assays

For in vitro studies, L929 fibroblast cells were cultured in DMEM medium (cat. no. 31600-083, Gibco, Dublin, Ireland) supplemented with 10% fetal bovine serum (FBS, cat. no. 10270-106, origin South America, Gibco, by Life Technologies, Carlsbad, CA, USA). The L929 cells were chosen for this study because this cell line is routinely used for biocompatibility testing of medical devices, as recommended in Ref [54]. The cell morphology was examined through optical microscopy and the cell viability and proliferation were evaluated quantitatively by MTT (3-(4,5-dimethylthiazol-2-yl)-2,5-diphenyltetrazolium bromide, Sigma–Aldrich Co, Steinheim, Germany) and LDH (lactate dehydrogenase, Tox7 kit, Sigma–Aldrich) assays.

MTT assay is a quantitative method used to evaluate cell-metabolic activity. Briefly, the cells were seeded in 24 well plates at a density of 1.5 × 10^4^ cells/mL in 500 µL culture medium. After exposure to hydrogel for 24 h, the culture medium was removed, replaced with a culture medium without fetal bovine serum (FBS) in which 1 mg/mL MTT solution was added. The absorbance of the samples was measured at 595 nm at a Flex Station 3 microplate reader (Molecular Devices, San Jose, CA, USA,) after the purple formazan formation and solubilization with 150 µL isopropanol.

The activity of LDH released into cell culture media was investigated using in vitro Toxicology Assay Kit, Lactic Dehydrogenase according to the manufacturer’s instructions. After exposure of the L929 cells for 24 h to hydrogels, the plates were incubated with a 50 µL reaction mix for 15 min at room temperature, in the dark, and the final solution was assessed by spectrophotometric measurement at 490 nm (Flex Station 3 microplate reader, Molecular Devices, San Jose, CA, USA).

The morphology of fibroblast cells was analyzed after 24 h of incubation with the control hydrogel, 2 mg/mL unirradiated CPZ-loaded hydrogel, and 2 mg/mL irradiated for 30 min CPZ-loaded hydrogel by using an Olympus IX73 microscope (Olympus, Tokyo, Japan) equipped with a Hamamatsu ORCA-03G camera (A3472-06, Hamamatsu, Japan) and phase-contrast images were acquired using CellSens Dimension software (v1.11, Olympus).

## 3. Results and Discussion

### 3.1. Hydrogel Synthesys

Solution of Irgacure at 0.05%, 0.35% and 0.7% *w*/*v* and GelMa 10% *w*/*v* were irradiated with 266 nm laser beams (at energies of 0.25, 0.45, 0.75 and 1 mJ) for 1, 5, 10, 20, and 30 min. For the mixture Irgacure at 0.7% and GelMa at 10% the hydrogels did not form, whereas for the mixture Irgacure at 0.35% and GelMa at 10% the hydrogels were partially crosslinked. Instead, for Irgacure at 0.05% and GelMa at 10%, the hydrogels were fully formed, except for the solutions exposed to 0.25 mJ for 1 and 5 min. Therefore, by increasing the photoinitiator concentration above 0.05%, the hydrogels were not completely formed because the concentration of Irgacure was too high on the surface, resulting in a decrease in the laser radiation’s penetration depth and an incomplete curing process. In this respect, this paper presents the investigation of the hydrogels formed from Irgacure at 0.05% and GelMa at 10%, hereinafter referred to as GelMa hydrogel.

Photopolymerization with pulsed laser radiation has several advantages such as a short curing time due to the possibility of changing the beam energy and of select excitation of chromophores because of the monochromatic light. Fluorescence emission has several advantages over other curing monitoring methods, including a fast response time, high sensitivity, and in situ non-invasive analysis [55,56,57]. In case of 365 nm UV lamps, longer exposure periods are needed for photopolymerization due to the low absorption cross section of Irgacure at this wavelength when compared with 266 nm. The advantage of the use of 266 nm laser beam radiations were the short exposure times due to the higher quantum efficiency of the photochemical and photophysical processes that took place in the excited state of Irgacure [58].

### 3.2. The Effect of Irradiation Time and Energy on the Photo-Crosslinking of the GelMa Hydrogels

#### 3.2.1. Unreacted Irgacure after Photo-Polymerization of GelMa Hydrogels Analysis

The amount of unreacted Irgacure after photo-polymerization was determined by direct measurement using UV-Vis absorption spectroscopy on the solutions resulting from 24 h immersion in water of the GelMa hydrogel photo-crosslinked when solutions of Irgacure (0.05%)-GelMa (10%) were exposed to 266 nm pulsed lasers at energies of 0.25, 0.45, 0.75, and 1 mJ for time intervals between 1 and 30 min. The unreacted Irgacure concentration was determined for each sample via the absorbance of the peak at 280 nm wavelength using a calibration curve determined from the absorption spectra measured for seven concentrations between 0.001 mg/Ml–0.3 mg/mL of Irgacure in ultrapure water.

**Figure 2:**
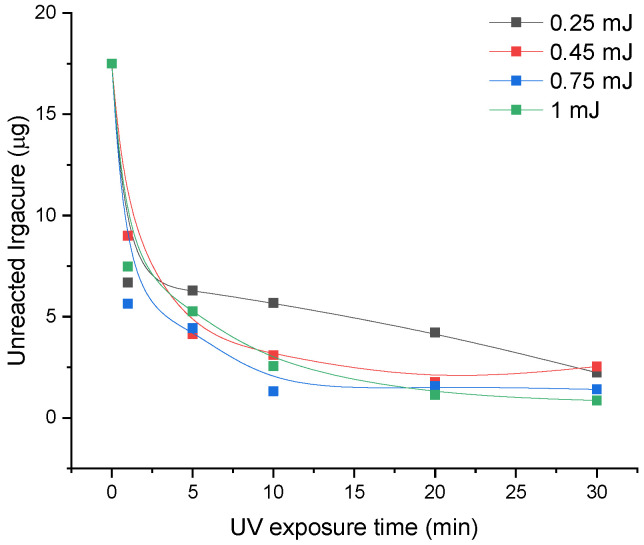
Influence of laser beam energy end exposure time on the unreacted Irgacure resulting after 24 h immersion in ultrapure water of the GelMa hydrogel photo-crosslinked when solutions of Irgacure (0.05%)-GelMa (10%) were exposed to 266 nm pulsed lasers at energies of 0.25, 0.45, 0.75, and 1 mJ for time intervals between 1 and 30 min. The amount of unreacted Irgacure was determined by direct measurement using UV–Vis absorption spectroscopy.

Knowing the concentration of unreacted Irgacure and the volume of water (3 mL) in which the hydrogel was immersed one can determine its quantity. The resulting quantities as a function of exposure time for laser energy between 0.25 mJ and 1 mJ are depicted in Figure 2. The initial amount of Irgacure used for the formation of hydrogels in the 35 µL volume was 17.5 µg.

The amount of unreacted Irgacure decreases abruptly in the first min for all the laser energies, where Irgacure-GelMa solution exposed to 0.75 mJ had the lowest value. Afterward, the decrease in unreacted Irgacure amounts is not so significant and have almost the same trend for all the investigated energies, with an exception in the case of 0.25 mJ where the quantities of unreacted Irgacure are higher than for the rest of energies.

#### 3.2.2. Swelling Behavior of GelMa Hydrogels

**Figure 3:**
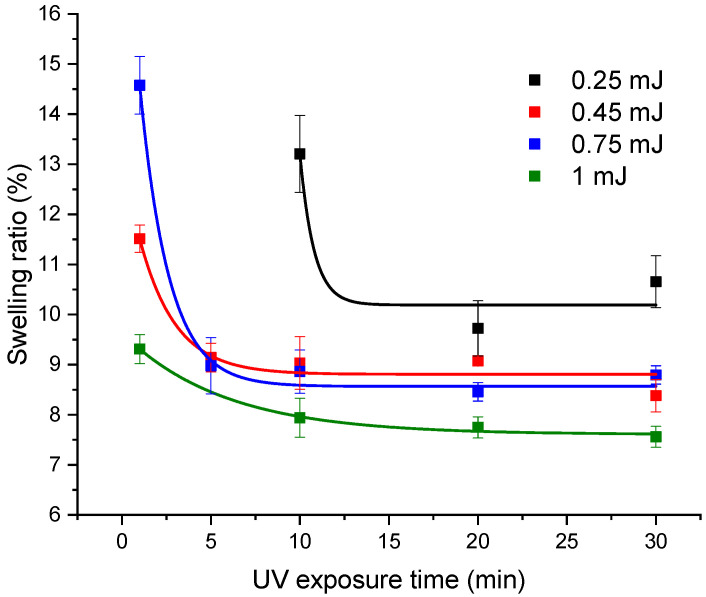
Influence of laser beam energy and exposure time on the swelling behavior of GelMa hydrogel photo-crosslinked when Irgacure (0.05%)-GelMa (10%) solutions were exposed 1, 5, 10, 20, 30 min to 0.25, 0.45, 0.75, and 1 mJ laser beams.

The swelling property of hydrogels is crucial because it affects the solute transport and their mechanical characteristics [24]. The degree of swelling is a balance between the forces that constrain the deformation of the network and the osmosis that leads to the water absorption and is influenced by the hydrogel structural characteristics, such as how they interaction with the solvent, the density of their crosslinks, and their hydrophilicity [59]. The swelling behavior of GelMa hydrogels photo-crosslinked up to 30 min at the four energies was investigated and is presented in Figure 3.

Because complete photo-crosslinking did not take place, the swelling ratios of the hydrogel that was created at 0.25 mJ for 1 min and 5 min exposure time are not present. The data, as shown in Figure 3, demonstrate how the swelling ratio is strongly influenced by UV laser beams’ energy and by the exposure time. When 0.75 mJ was used instead of 0.45 mJ for one-min exposure time, the swelling properties improved. However, when 1 mJ beams were applied, the swelling ratio decreased in comparison to the lower energies. This indicates that energies of 1 mJ have a detrimental effect on the mechanical properties of the hydrogels by decreasing the swelling due to the increase in crosslinking degree resulting from higher energy intensity. The characteristics of the hydrogels are similarly impacted by exposure times greater than 1 min, with the swelling ratio significantly lower than at 1 min. This observation is consistent with the hydrogels’ changing color. The hydrogels began to turn yellow after 5 min of exposure at 0.45 and 0.75 mJ beams and, as the exposure time increased, so did the color, which changed to a dark yellow. The hydrogels began to turn yellow for 0.25 mJ after 20 min and for 1 mJ after 1 min. This coloring is perhaps due to photo-oxidation processes that are produced by excess energy not used in the photo-crosslinking process [60].

#### 3.2.3. In Vitro Release of CPZ from the GelMa Hydrogels

**Figure 4:**
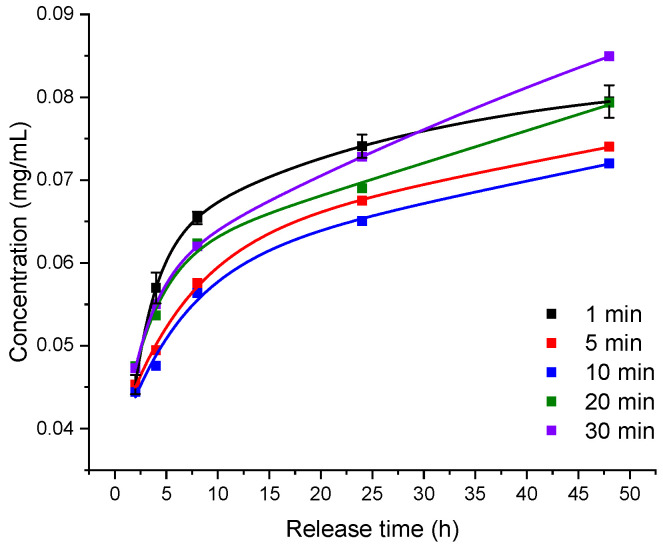
Influence of exposure time on the in vitro CPZ release profile from GelMa hydrogels resulting from 266 nm laser exposure up to 30 min at 0.75 mJ beam energy, as measured in PBS solution at 37 °C.

The in vitro drug release results are presented in Figure 4. UV–Vis absorption spectroscopy was used to quantify the amounts of released CPZ. The analyses of in vitro release data suggest a linear burst release within the first 8 h, followed by a slower continuous release of the CPZ over more than 40 h. The initial burst release may be partly caused by CPZ particles attached to the surface of the hydrogels that are highly susceptible to diffusion.

The irradiation time influences the drug release in such a way that the amount of released CPZ is decreasing with the increase in the irradiation time. In other words, the hydrogels resulting from 1 min laser exposure showed more sustained release than the ones obtained at longer exposure times.

The release profile of irradiated CPZ is difficult to quantify because the affinity of the photoproducts to bind to the network chains of the hydrogel is not known, thus the concentration of each photoproduct loaded into hydrogels is unknown.

Taking into account the results on unreacted Irgacure, swelling behavior, and CPZ release, as well as the transparency of the studied hydrogels, the best conditions for obtaining hydrogels were found to be 0.75 mJ laser beam energy and 1 min exposure time. Further, the subsequent studies only discuss the hydrogels that were produced under these favorable conditions.

### 3.3. Characterization of the GelMa Hydrogels Resulting when 0.75 mJ Beam Energy and 1 min Exposure Time Were Used

There have been multiple investigations on Irgacure 2959 as a photoinitiator in the photopolymerization using 365 nm emitting lamps of various polymers, such as poly(ethylene glycol) diacrylate [61,62], gelatin-methacryloyl [27,28], or methacrylate hyaluronic acid [27]. These studies provided hydrogel characterization without presenting the optical photoinitiator’s properties. By focusing on the photoinitiator, during UV light exposure, the hydrogel-forming process can be improved and the number of trial-and-error tests can be reduced.

#### 3.3.1. Laser-Induced Fluorescence and Fluorescence Kinetics Profile Assay

**Figure 5:**
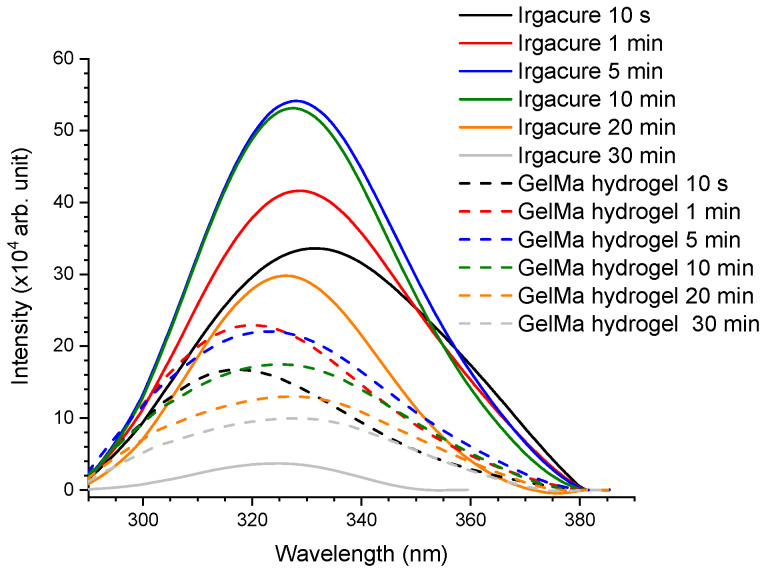
LIF spectra of Irgacure (0.05% *w*/*v*) and GelMa hydrogel (Irgacure 0.05% and GelMa 10% *w*/*v*) exposed to 266 nm laser pulsed radiation at an energy of 0.75 mJ up to 30 min.

The laser-induced fluorescence (LIF) was collected in real-time during the irradiation of Irgacure and Irgacure-GelMa. This method had the benefit of monitoring in real-time the photocleavage of the Irgacure molecule. A LIF spectrum was collected for each 50 excitation laser pulses after each 5 s, respectively. This method offered both the fluorescence spectrum and the fluorescence kinetics profile of irradiated Irgacure. For each irradiation time of 1, 5, 10, 20, and 30 min, the fluorescence spectra are shown in Figure 5. The LIF spectrum of Irgacure is characterized by one band with a peak at 330 nm and that of GelMa hydrogel has a peak at 317 nm, respectively. For Irgacure, the wavelength of fluorescence peak was blue-shifted to 328 nm after 1 min and remained unchanged up to 10 min of UV exposure. After 10 min of irradiation, the fluorescence intensity started to decrease until it was almost quenched after 30 min. For GelMa hydrogel, the peak wavelength experienced a red-shift to 320 nm after the first min and it continued to do so up to 329 nm after 30 min of laser irradiation. Additionally, the fluorescence intensity started to decrease after 5 min of exposure.

**Figure 6:**
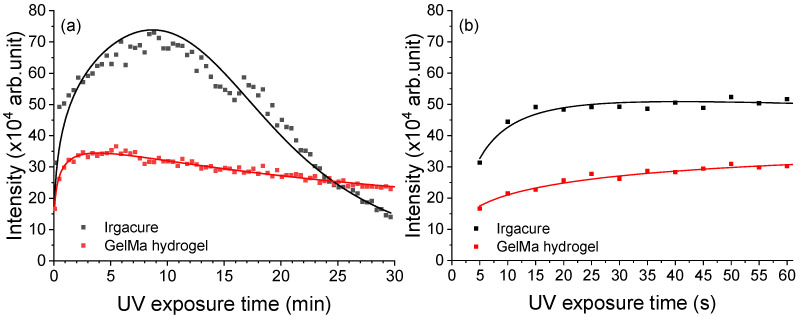
Fluorescence kinetics profile of Irgacure (0.05% *w*/*v*) and GelMa hydrogel (Irgacure 0.05% and GelMa 10% *w*/*v*) exposed to 266 nm laser beams at an energy of 0.75 mJ for (**a**) 30 min and (**b**) 1 min.

Further, the fluorescence kinetics profiles of Irgacure and GelMa hydrogel exposed to 266 nm laser beams for 30 min were investigated and are presented in Figure 6a. Figure 6b shows the modification induced by the radiation in the first min or more specifically when the hydrogel formed. The LIF intensity varied depending on the irradiation period. The LIF signal for Irgacure reached an intensity maximum after 530 s and almost vanished (indicating photocleaving) after 30 min, whereas GelMa hydrogel showed an increase in the first 155 s followed by a slight and constant decrease.

The changes in fluorescence intensity of Irgacure and GelMa hydrogel when exposed to UV light are influenced by radical production, molecular oxygen consumption, or radical recombination into the parental molecule. The cleavage of Irgacure in the liquid phase leads to two radicals that exist next to each other for a certain time surrounded by solvent, the so-called “cage effect” [63]. Due to the high-rate coefficient for the interaction of the two radicals, they recombine before they dissociate by diffusion being susceptible once again to fluorescence emission.

The presence of molecular oxygen, which can hinder the polymerization process by extinguishing the excited states of the initiator, is a factor that limits the usage of photopolymerization with radicals [64,65], even though this method is commonly employed in biological applications [66,67]. The consumption of molecular oxygen can be monitored by laser-induced fluorescence. The presence of molecular oxygen can be accounted in the fluorescence spectra, Figure 6a, by an induction period observed in the spectra as the oxygen quenches the Irgacure fluorescence. This induction period represents the consumption of oxygen by the radicals [55]. For GelMa hydrogel a shorter induction period was observed because the radicals were involved and consumed in the crosslinking of the hydrogel. The fluorescence intensity of GelMa hydrogel decreased by 50% when compared to that of Irgacure (Figure 6b). This difference in fluorescence intensity is because of Irgacure consumption during hydrogel curing and the stiffness of the surrounding environment as the GelMa is crosslinked. Thus, an important aspect is the necessity to have an online method to monitor the curing of the hydrogels. Currently a visual inspection is made to assess that the hydrogels are formed, followed by their characterization using various off-line methods. In this respect, LIF measurements have the advantage that they can be used successfully to monitor online the hydrogel photo-crosslinking.

#### 3.3.2. FTIR Spectroscopy

**Figure 7:**
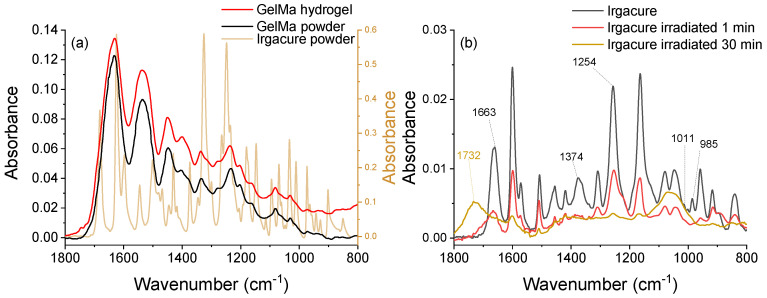
FTIR spectra of (**a**) Irgacure 2959 powder, GelMa powder, and dried GelMa hydrogel (Irgacure 0.05% and GelMa 10% *w*/*v*) resulting after the irradiation with 0.75 mJ pulsed laser beams for 1 min, (**b**) Irgacure at concentration 0.05% exposed to 266 nm laser pulsed radiation at energy 0.75 mJ for 1 min and 30 min.

When the photoinitiator concentration and UV radiation exposure periods are varied, FTIR spectroscopy provides excellent insight into the reactions as function of the changed parameters. The IR spectra of dried GelMa hydrogel were compared with that of Irgacure and GelMa, powder form, and are shown in Figure 7a. Additionally, the FTIR spectra of Irgacure irradiated for 1 and 30 min Irgacure are presented in Figure 7b.

The IR spectra of GelMa hydrogel showed the characteristic vibration of GelMa as follows: 1630 cm^− 1^—stretching vibration C=O (amide I), 1544 cm^−1^—deformation vibration of C–N–H, 1237 cm^−1^—deformation vibration N–H (amide II), 1450 cm^−1^—deformation vibration C–H, 1335 cm^−1^—C–N stretching vibration within amide III. Thus, the hydrogels did not show any significant change in the structure of the polymer during the photopolymerization process. Irgacure peaks were not visible in the GelMa hydrogel IR spectrum because Irgacure concentration was 200 times lower than that of GelMa prior photo-crosslinking and even lower after the unreacted molecules were removed during the 24 h immersion in water. 

In the context of the GelMa hydrogel photopolymerization, it is necessary to understand how Irgacure cleaves into radicals when exposed to 266 nm-pulsed laser beams to help in optimizing the photo-crosslinking.

To analyze the photocleavage pathway, the laser-exposed solutions were compared to the unirradiated Irgacure solution. The IR spectra of 0.05% Irgacure (Figure 7b) show the characteristic IR vibrations of Irgacure in accordance with the literature [68,69] and presented a significant difference between unirradiated and 1 min irradiated samples. As a result, the band with the peak at 1663 cm^−1^ (C=O stretching vibration) widened, while the bands at 1374 cm^−1^ (C−H wagging vibration, CH_2_) and 1011 cm^−1^ (C−H wagging vibration, CH_3_) disappeared. The band with peak at 985 cm^−1^ (C–H out-of-plane vibration, phenyl ring) also faded away. Moreover, the remaining bands were shifted with 2 cm^−1^ to higher wavenumbers. After 5 min irradiation, the band at 1663 cm^−1^ continued to be shifted to higher wavenumbers. With more irradiation times, major changes in the IR spectra were observed: the band at 1663 cm^−1^ was shifted to 1732 cm^−1^, the peak situated between 1490–1290 cm^−1^ disappeared, the bands with peaks at 1080 and 1045 cm^−1^ vanished, and a new peak at 1065 cm^−1^ appeared. These changes suggested the total photocleavage of Irgacure.

The photo-fragmentation of Irgacure is explained by the Norrish type I reaction. The cleavage occurs at α-cleavage of a C–C bond into benzoyl and hydroxyalkyl radicals via the excited singlet or triplet state [66,67], which further leads to a set of fragmentation reactions based on the movement of those radicals by diffusion and rotation. Another possibility is the recombination of the two radicals in Irgacure inside the solvent cage. The polymerization process could be initiated by one or both radicals.

The disappearance of the band with the peak at 1663 cm^−1^ and appearance at 1725 cm^−1^ was the first indicator that Irgacure photo-cleaved. Both peaks are attributed to C=O stretching vibration, the longer wavenumbers band appearance suggested the cleavage of the α-carbon from the ketone group. This was also confirmed by the near-disappearance of the band with peak at 1254 cm^−1^ (C–C–C skeletal vibration from aromatic ketone). Additionally, the bands responsible for C–H vibration and C–C skeletal vibration from C–CH_3_, such as 1374, 1011, and 985 cm^−1^ supported the photo-cleavage of Irgacure molecule photocleaved into benzoyl and hydroxyalkyl radicals.

#### 3.3.3. UV-Vis Sbsorption Spectroscopy

**Figure 8:**
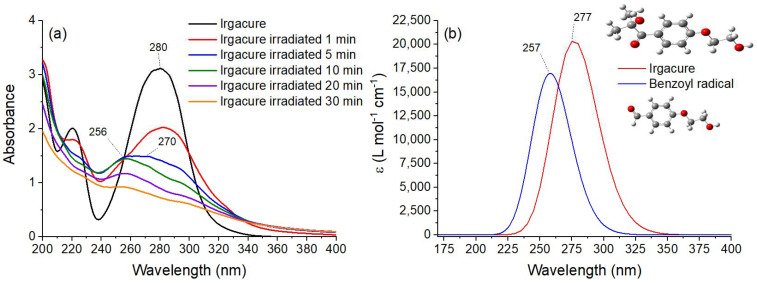
(**a**) UV–Vis absorption spectra of Irgacure (0.05% *w*/*v*) exposed to 266 nm laser pulsed radiation at an energy of 0.75 mJ up to 30 min; (**b**) UV–Vis spectra of Irgacure and benzoyl radical resulting from DFT B3LYP 6-311(d,p) computation with Gaussian 09.

To confirm the IR measurements regarding the generation of benzoyl and hydroxyalkyl radicals, the Irgacure solutions exposed to 266 nm pulsed laser were subjected to UV–Vis absorptions spectroscopy assays and the absorption spectra are shown in Figure 8a. The UV–Vis spectrum of Irgacure shows two absorption bands with peaks at 220 and 280 nm. The absorbance band with a peak at 280 nm decreased in the first min. After 5 min of exposure, the band at 280 nm disappeared and a new broadband with peak at 270 nm appeared, followed by its disappearance and appearance of a new band with peak at 256 nm after 10 min. The spectrum after 30 min irradiation showed the complete photocleavage of Irgacure.

The changes in the UV–Vis absorption spectra of Irgacure exposed to 266 nm laser beams suggested the photo-decomposition into radicals as observed in IR spectra (Figure 7). It is proposed that by absorbing a UV photon, the molecule is excited in the singlet state and by intersystem-crossing transition is transferred to a triplet state, and from this state, benzoyl and hydroxyalkyl radicals are formed [58]. In this respect, Gaussian 09 software was used to optimize the structures of Irgacure and benzoyl radical and to simulate their absorption spectra for comparison purposes. The calculated UV–Vis spectra of Irgacure and benzoyl groups are presented in Figure 8b. When compared to Irgacure, the peak of the benzoyl radical was blue-shifted to 258 nm. The same blue shift was observed in the UV–Vis experimental spectra of irradiated Irgacure compared with that of the unirradiated (Figure 8a). Thus, UV–Vis spectra confirmed the degradation of Irgacure into benzoyl radical during exposure to 266 nm laser beams.

Also, the presence of isosbestic points in the UV–Vis spectra (Figure 8a) after 1 min of irradiation confirmed the formation of radicals with absorption bands overlapped on Irgacure absorption spectrum [70].

#### 3.3.4. SEM Snalysis

**Figure 9:**
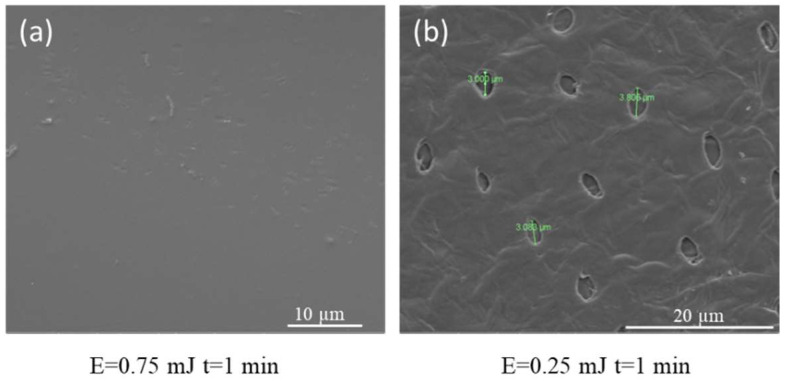
SEM images of GelMa hydrogels (Irgacure 0.05% and GelMa 10% *w*/*v*) obtained after 1 min UV-pulsed laser radiation exposure at energies of (**a**) 0.75 mJ and (**b**) 0.25 mJ.

SEM provides information about the structure and composition of the sample and other properties such as electrical conductivity. It can capture the characteristic network structure of hydrogels by determining the hydrogel porosity. Figure 9 shows the SEM images obtained for the dry GelMa hydrogels obtained after 1 min UV-pulsed laser radiation exposure at energies of 0.75 mJ and 0.25 mJ, respectively.

The SEM images show, in Figure 9a, that the hydrogel obtained from 1 min laser exposure at 0.75 mJ do not possess physical pores; instead they have a smooth and homogeneous morphological structure. The interconnected structure of hydrogels gives them specific properties such as the ability not to dissolve in a solvent and the capacity to retain water. By comparison, Figure 9b shows the image of hydrogels obtained at 0.25 mJ for 1 min. The holes with a diameter of ~3 µm are indications of the partial formation of this hydrogel.

### 3.4. In Vitro Biological Evaluation of Unirradiated and Irradiated CPZ-Loaded GelMa Hydrogels

#### 3.4.1. Disc Diffusion Sensitivity Assay of Loaded Hydrogels against *S. aureus* and MRSA

**Figure 10:**
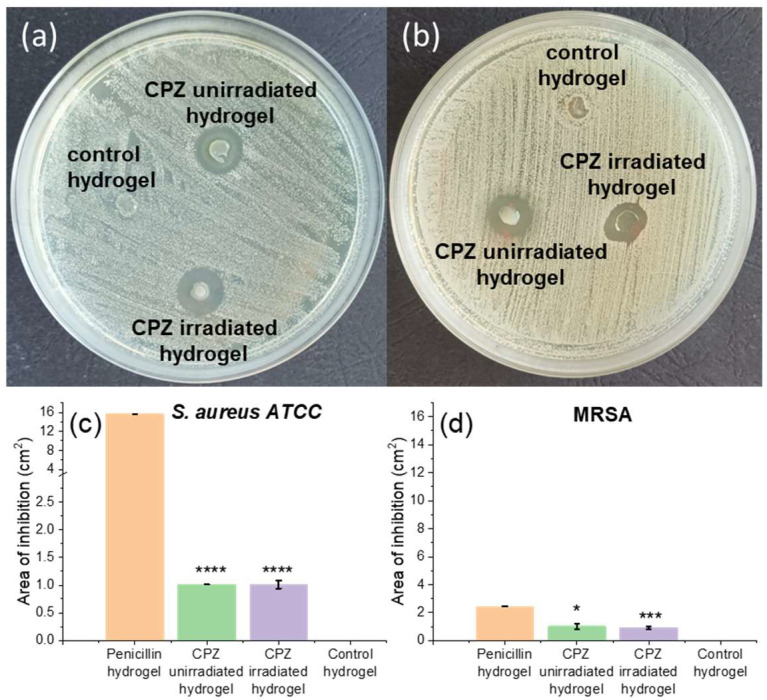
Disc diffusion sensitivity test of unloaded GelMa hydrogel and GelMa hydrogels loaded with 2 mg/mL unirradiated CPZ and 2 mg/mL irradiated for 30 min CPZ against (**a**) *S. aureus*, (**b**) MRSA. Area of inhibition and corresponding standard deviation of the GelMa hydrogels loaded with penicillin (positive control), unirradiated and irradiated CPZ against (**c**) *S. aureus* and (**d**) MRSA strains. Data expressed as mean ± SD, *n* = 3, data points analyzed by *t*-test, levels of statistical significance between the penicillin and unirradiated/irradiated CPZ groups of **** *p* < 0.0001, * *p* < 0.05, *** *p* < 0.0005.

Agar disk-diffusion testing was used in this study to evaluate the antimicrobial activity of the hydrogels loaded with unirradiated CPZ and 30 min irradiated CPZ against *S. aureus* ATCC 25923 and MRSA, known to be frequently responsible for skin infections [43,44]. After 18 h incubation at 37 °C, the unirradiated and irradiated CPZ-loaded hydrogels inhibited *S. aureus* (Figure 10a) and MRSA (Figure 10b) growth.

Whereas the area of inhibition, determined using ImageJ software [71] and produced by CPZ unirradiated loaded hydrogel, was comparable to that of CPZ irradiated loaded hydrogel for *S. aureus* and slightly higher for MRSA (Figure 10c), growth of bacteria can be seen in Figure 10a,b within the area of inhibition of CPZ unirradiated, and more significantly for MRSA. Therefore, irradiated CPZ exhibits better antibacterial activity than unirradiated CPZ.

#### 3.4.2. Bacterial Adherence to Loaded GelMa Hydrogels Assay

**Figure 11:**
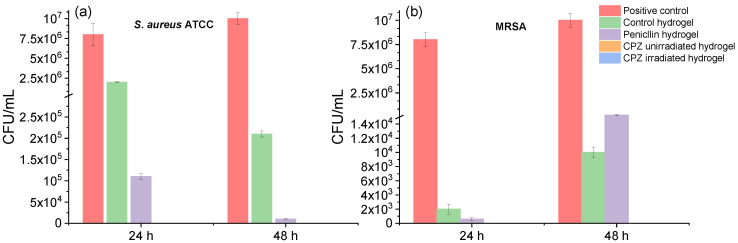
Colony forming unit (CFU) analysis of (**a**) *S. aureus* ATCC and (**b**) MRSA after 24 and 48 h treatment with hydrogels loaded with unirradiated CPZ, 30 min irradiated CPZ, and penicillin; the positive control is represented by pure bacterial culture in the absence of hydrogels; data expressed as mean ± SD, *n* = 3.

Further, the *S. aureus* and MRSA bacterial adherence to unirradiated and irradiated CPZ-loaded hydrogels were determined through the viable cell counts method over a 48 h incubation period. In the case of *S. aureus*, as seen in Figure 11a, the bacteria colonies attached to hydrogels loaded with unirradiated/irradiated CPZ were completed inhibited, even after 48 h. On the other hand, the 2 mg/mL penicillin loaded hydrogel was unable to inhibit the standard *S. aureus* strain (10^5^ CFU/mL), however, its CFU/mL value decreased after 48 h and reached 10^4^ CFU/mL. Furthermore, after we observed the effect against the standard strain of the unirradiated and laser-exposed medicine hydrogels, the same drug was used to demonstrate the efficiency across antibiotic-resistant bacteria, MRSA, and both unirradiated CPZ and irradiated CPZ hydrogels prevented the development of the microorganism around the loaded hydrogels (Figure 11b). Instead, for penicillin, the number of MRSA colonies was smaller after 24 h (4 × 10^2^ CFU/mL) than after 48 h (15 × 10^4^ CFU/mL). Therefore, both unirradiated and irradiated CPZ were more effective than penicillin at preventing the adhesion of *S. aureus* and MRSA to hydrogels.

#### 3.4.3. In Vitro Evaluation of L929 Cells’ Viability and Morphology

When developing oral or wound-dressing materials, biocompatibility and the absence of cytotoxicity are important properties that need to be fulfilled. The influence of unirradiated and irradiated CPZ-loaded hydrogels on cells’ viability and membrane integrity was established by in vitro methods (MTT and LDH) assessed on normal fibroblasts. Morphological modifications were also investigated by optical microscopy.

**Figure 12:**
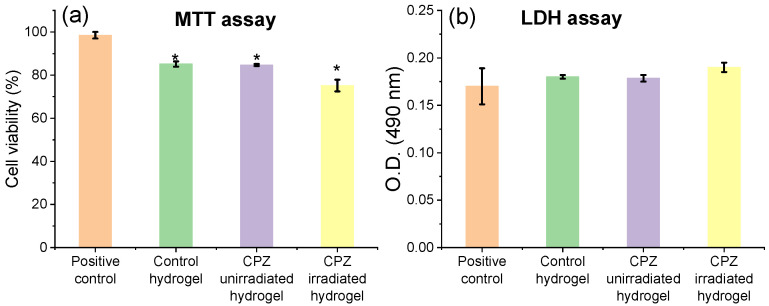
(**a**) In vitro L929 cell viability after 24 h incubation with the hydrogel loaded with 2 mg/mL unirradiated CPZ, and the hydrogel loaded with 2 mg/mL irradiated for 30 min CPZ; (**b**) LDH release after membrane damage of L929 cells incubated with samples for 24 h; the positive control represents L929 cell in standard culture conditions in the absence of hydrogels; data expressed as mean ± SD, *n* = 3, data points analyzed by *t*-test, levels of statistical significance between the analyzed groups of * *p* ≤ 0.05.

The results are presented in Figure 12a for the MTT assay and in Figure 12b for LDH assay. Two different CPZ-loaded hydrogels (unirradiated and irradiated, having a concentration of 2 mg/mL) were tested to verify the non-cytotoxic effect on cells after 24 h incubation. The MTT evaluation shows that the cell viability is not significantly affected by the tested biomaterial, the cytotoxicity of the hydrogel loaded with irradiated CPZ was up to 76%, compared with unirradiated (86%) or the hydrogel control (86.3%) as observed in Figure 12a. According to ISO EN 10993-5 protocol, the cytotoxic potential of a sample is recognized when the viability value of cells in contact with a sample is less than 70% of the negative control [72]. Therefore, it can be stated that the hydrogels loaded with unirradiated and irradiated CPZ did not present a cytotoxic effect on L929 cells.

In addition, the LDH released in culture media was also measured to estimate the cell membrane damage after 24 h incubation with the hydrogels loaded with unirradiated and irradiated CPZ. As shown in Figure 12b, loss of cell membrane integrity was present for the loaded hydrogels and control hydrogel, but not significant compared with the cell control, the level of LDH increased by a maximum of 5% for the hydrogel loaded with unirradiated CPZ and 11.7% for the hydrogel loaded with irradiated CPZ, which suggests that the loaded hydrogels succeeded in keeping the cell membrane integrity after 24 h of exposure to samples.

**Figure 13:**
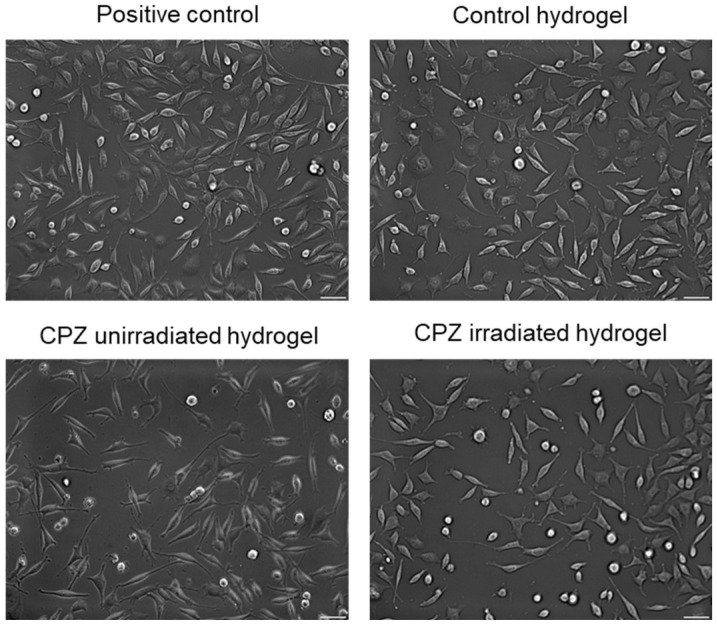
Bright-field images of L929 cells morphology after 24 h exposure to 2 mg/mL unirradiated CPZ and 2 mg/mL irradiated CPZ for 30 min loaded hydrogels in comparison with the positive control; the positive control represents L929 cell in standard culture conditions in the absence of hydrogels; scale set at 20µm.

Figure 13 represents the morphological observations that reveal intact cell membrane from control to hydrogels. Therefore, the L929 fibroblasts maintained their fibroblast-specific elongated morphology after 24 h exposure to unirradiated/irradiated CPZ-loaded hydrogels with no significant differences between the samples compared to the control.

Thus, the CPZ unirradiated/irradiated loaded GelMa hydrogels do not significantly affect the viability of L929 cells and do not stimulate proliferation. If they did, one would have seen a difference in the MTT test, respectively a higher cell viability percentage for the loaded GelMa hydrogels.

Since the direct contact of CPZ-loaded hydrogels with the L929 cells did not indicate any changes in the cell morphology and proliferation, the hydrogels loaded with unirradiated CPZ and irradiated CPZ can be considered to be biocompatible.

## 4. Conclusions

In conclusion, GelMa hydrogels loaded with unirradiated CPZ and irradiated CPZ with antimicrobial and non-cytotoxic properties were obtained using 266 nm UV-pulsed laser radiation instead of a UV lamp. To find the ideal hydrogel in terms of water-holding capacity, permeability, drug release, antimicrobial activity, and biocompatibility, different Irgacure concentrations and pulsed UV laser energies and exposure time intervals were tested. As a result, when GelMa concentration was 10% *w*/*v*, the ideal conditions for Irgacure were at a concentration of 0.05% *w*/*v* and a UV exposure time of 1 min. Irgacure fluorescence and its fluorescence kinetics profile were used to monitor how the radicals were attached to the polymer backbones.

Also, GelMa hydrogels showed a prolonged drug release profile over 48 h. The amount of released unirradiated/irradiated CPZ is high enough to suppress bacterial growth, both on the surface of the nutrient agar and in the liquid medium, without affecting the morphology and viability of normal healthy cells. In this regard, it appears promising to use 266 nm UV-pulsed laser radiation to photo-crosslink GelMa hydrogels, which can be used as drug delivery systems for skin-infected wounds.

Considering GelMa hydrogels as potential drug delivery systems of irradiated CPZ for the treatment of skin-infected wound, performing more cytotoxicity assays on representative cell lines for the outer layer of the skin such as keratinocytes, melanocytes or Langerhans cells would also be appropriate. 

## Data Availability

Data available on request.

## References

[1] Cohen J. (1995). Clinical trials: IL-12 deaths: Explanation and a puzzle. Science.

[2] Florence A.T., Jani P.U. (1994). Novel oral drug formulations: Their potential in modulating adverse effects. Drug Saf..

[3] Roy R., Tiwari M., Donelli G., Tiwari V. (2018). Strategies for combating bacterial biofilms: A focus on anti-biofilm agents and their mechanisms of action. Virulence.

[4] Arjama M., Mehnath S., Rajan M., Jeyaraj M. (2018). Sericin/RBA embedded gellan gum based smart nanosystem for PH responsive drug delivery. Int. J. Biol. Macromol..

[5] Mehnath S., Chitra K., Karthikeyan K., Jeyaraj M. (2020). Localized delivery of active targeting micelles from nanofibers patch for effective breast cancer therapy. Int. J. Pharm..

[6] Hussain K., Aslam Z., Ullah S., Shah M.R. (2021). Synthesis of PH responsive, photocrosslinked gelatin-based hydrogel system for control release of ceftriaxone. Chem. Phys. Lipids.

[7] Ribeiro J.S., Sanz C.K., Münchow E.A., Kalra N., Dubey N., Suárez C.E.C., Fenno J.C., Lund R.G., Bottino M.C. (2022). Photocrosslinkable methacrylated gelatin hydrogel as a cell-friendly injectable delivery system for chlorhexidine in regenerative endodontics. Dent. Mater..

[8] Walter J.R., Xu S. (2015). Therapeutic transdermal drug innovation from 2000 to 2014: Current status and outlook. Drug Discov. Today.

[9] Prausnitz M.R., Langer R. (2008). Transdermal drug delivery. Nat. Biotechnol..

[10] Almoshari Y. (2022). Novel hydrogels for topical applications: An updated comprehensive review based on source. Gels.

[11] Lim H.W., Collins S.A.B., Resneck J.S., Bolognia J.L., Hodge J.A., Rohrer T.A., Van Beek M.J., Margolis D.J., Sober A.J., Weinstock M.A. (2017). The burden of skin disease in the United States. J. Am. Acad. Dermatol..

[12] Dawson A.L., Dellavalle R.P., Elston D.M. (2012). Infectious skin diseases: A review and needs assessment. Dermatol. Clin..

[13] Dannaker C. (2015). Waterborne Topical Compositions for the Delivery of Azelaic Acid for Treatment of Skin Conditions Such as Acne Vulgaris, Rosacea Seborrheic Dermatitis. U.S. Patent.

[14] Xia Z., Marsh A. (2013). Topical Drug Patch Including Microspheres. U.S. Patent.

[15] Sachdeva M.S., Patlolla R. (2009). Nanoparticle Formulations for Skin Delivery. U.S. Patent.

[16] Zhang Y., Hansen K.J., Determan A.S. (2012). Microneedle Devices and Methods. AU Patent.

[17] Narayanaswamy R., Torchilin V.P. (2019). Hydrogels and their applications in targeted drug delivery. Molecules.

[18] Gupta B., Agarwal R., Alam M.S. (2011). Hydrogels for wound healing applications. Biomedical Hydrogels.

[19] Li J., Mooney D.J. (2016). Designing hydrogels for controlled drug delivery. Nat. Rev. Mater..

[20] Almeida J.F., Ferreira P., Lopes A., Gil M.H. (2011). Photocrosslinkable biodegradable responsive hydrogels as drug delivery systems. Int. J. Biol. Macromol..

[21] Kamoun E.A., Winkel A., Eisenburger M., Menzel H. (2016). Carboxylated camphorquinone as visible-light photoinitiator for biomedical application: Synthesis, characterization, and application. Arab. J. Chem..

[22] Burke G., Cao Z., Devine D.M., Major I. (2019). Preparation of biodegradable polyethylene glycol dimethacrylate hydrogels via thiol-ene chemistry. Polymers.

[23] Sabnis A., Rahimi M., Chapman C., Nguyen K.T. (2009). Cytocompatibility studies of an in situ photopolymerized thermoresponsive hydrogel nanoparticle system using human aortic smooth muscle cells. J. Biomed. Mater. Res..

[24] Nichol J.W., Koshy S.T., Bae H., Hwang C.M., Yamanlar S., Khademhosseini A. (2010). Cell-laden microengineered gelatin methacrylate hydrogels. Biomaterials.

[25] Loessner D., Meinert C., Kaemmerer E., Martine L.C., Yue K., Levett P.A., Klein T.J., Melchels F.P.W., Khademhosseini A., Hutmacher D.W. (2016). Functionalization, preparation and use of cell-laden gelatin methacryloyl—Based hydrogels as modular tissue culture platforms. Nat. Protoc..

[26] Tomal W., Ortyl J. (2020). Water-soluble photoinitiators in biomedical applications. Polymers.

[27] Zhang Y.S., Davoudi F., Walch P., Manbachi A., Luo X., Dell’Erba V., Miri A.K., Albadawi H., Arneri A., Li X. (2016). Bioprinted thrombosis-on-a-chip. Lab Chip.

[28] Assmann A., Vegh A., Ghasemi-Rad M., Bagherifard S., Cheng G., Sani E.S., Ruiz-Esparza G.U., Noshadi I., Lassaletta A.D., Gangadharan S. (2017). A highly adhesive and naturally derived sealant. Biomaterials.

[29] Williams C.G., Malik A.N., Kim T.K., Manson P.N., Elisseeff J.H. (2005). Variable cytocompatibility of six cell lines with photoinitiators used for polymerizing hydrogels and cell encapsulation. Biomaterials.

[30] Denmark D.J., Hyde R.H., Gladney C., Phan M.-H., Bisht K.S., Srikanth H., Mukherjee P., Witanachchi S. (2017). Photopolymerization-based synthesis of iron oxide nanoparticle embedded PNIPAM nanogels for biomedical applications. Drug Deliv..

[31] Qin X.-H., Ovsianikov A., Stampfl J., Liska R. (2014). Additive manufacturing of photosensitive hydrogels for tissue engineering applications. BioNanoMaterials.

[32] Klotz B.J., Gawlitta D., Rosenberg A.J.W.P., Malda J., Melchels F.P.W. (2016). Gelatin-methacryloyl hydrogels: Towards biofabrication-based tissue repair. Trends Biotechnol..

[33] O’Connell C.D., Zhang B., Onofrillo C., Duchi S., Blanchard R., Quigley A., Bourke J., Gambhir S., Kapsa R., Di Bella C. (2018). Tailoring the mechanical properties of gelatin methacryloyl hydrogels through manipulation of the photocrosslinking conditions. Soft Matter.

[34] Billiet T., Gevaert E., De Schryver T., Cornelissen M., Dubruel P. (2014). The 3D printing of gelatin methacrylamide cell-laden tissue-engineered constructs with high cell viability. Biomaterials.

[35] Ouyang L., Highley C.B., Sun W., Burdick J.A. (2017). A Generalizable strategy for the 3D bioprinting of hydrogels from nonviscous photo-crosslinkable inks. Adv. Mater..

[36] Vigata M., Meinert C., Pahoff S., Bock N., Hutmacher D.W. (2020). Gelatin methacryloyl hydrogels control the localized delivery of albumin-bound paclitaxel. Polymers.

[37] Zhu M., Wang Y., Ferracci G., Zheng J., Cho N.-J., Lee B.H. (2019). Gelatin methacryloyl and its hydrogels with an exceptional degree of controllability and batch-to-batch consistency. Sci. Rep..

[38] Alexandru T., Staicu A., Pascu A., Radu E., Stoicu A., Nastasa V., Dinache A., Boni M., Amaral L., Pascu M.L. (2014). Characterization of mixtures of compounds produced in chlorpromazine aqueous solutions by ultraviolet laser irradiation: Their applications in antimicrobial assays. J. Biomed. Opt..

[39] Alexandru T., Armada A., Danko B., Hunyadi A., Militaru A., Boni M., Nastasa V., Martins A., Viveiros M., Pascu M. (2013). Biological evaluation of products formed from the irradiation of chlorpromazine with a 266 nm laser beam. Biochem. Pharmacol..

[40] Armada A.M., Alexandru T., Machado D., Danko B., Hunyadi A., Dinache A., Nastasa V., Boni M., Ramos J., Viveiros M. (2013). The in vitro activity of products formed from exposure of chlorpromazine to a 266 nm laser beam against species of mycobacteria of human interest. In Vivo.

[41] Tozar T., Nastasa V., Stoicu A., Chifiriuc M.C., Popa M., Kamerzan C., Pascu M.L. (2019). In vitro antimicrobial efficacy of laser exposed chlorpromazine against gram-positive bacteria in planktonic and biofilm growth state. Microb. Pathog..

[42] Nistorescu S., Gradisteanu Pircalabioru G., Udrea A.-M., Simon A., Pascu M.L., Chifiriuc M.-C. (2020). Laser-irradiated chlorpromazine as a potent anti-biofilm agent for coating of biomedical devices. Coatings.

[43] Giudice P. (2020). Skin infections caused by staphylococcus aureus. Acta Derm. Venerol..

[44] Montgomery C.P., David M.Z., Daum R.S. (2015). Host factors that contribute to recurrent staphylococcal skin infection. Curr. Opin. Infect. Dis..

[45] Nagasawa N., Yagi T., Kume T., Yoshii F. (2004). Radiation crosslinking of carboxymethyl starch. Carbohydr. Polym..

[46] Spiller K.L., Laurencin S.J., Lowman A.M. (2009). Characterization of the behavior of porous hydrogels in model osmotically-conditioned articular cartilage systems. J. Biomed. Mater. Res..

[47] Gustafson C.T., Boakye-Agyeman F., Brinkman C.L., Reid J.M., Patel R., Bajzer Z., Dadsetan M., Yaszemski M.J. (2016). Controlled delivery of vancomycin via charged hydrogels. PLoS ONE.

[48] Frisch M.J., Trucks G.W., Schlege H.B., Scuseria G.E., Robb M.A., Cheeseman J.R., Scalmani G., Barone V., Petersson G.A., Nakatsuji H. (2016). Gaussian 09.

[49] Cossi M., Barone V., Cammi R., Tomasi J. (1996). Ab initio study of solvated molecules: A new implementation of the polarizable continuum model. Chem. Phys. Lett..

[50] Brown D.F., Kothari D. (1975). Comparison of antibiotic discs from different sources. J. Clin. Pathol..

[51] Bauer A.W. (1959). Single-disk antibiotic-sensitivity testing of staphylococci: An analysis of technique and results. AMA Arch. Intern. Med..

[52] Dutta D., Willcox M. (2013). A laboratory assessment of factors that affect bacterial adhesion to contact lenses. Biology.

[53] Lima M., Teixeira-Santos R., Gomes L.C., Faria S.I., Valcarcel J., Vázquez J.A., Cerqueira M.A., Pastrana L., Bourbon A.I., Mergulhão F.J. (2021). Development of chitosan-based surfaces to prevent single- and dual-species biofilms of staphylococcus aureus and pseudomonas aeruginosa. Molecules.

[54] Technical Committee (2009). ISO/TC 194 part 12: Preparation of samples and reference materials. ISO 10993-5:2009 Biological Evaluation of Medical Devices.

[55] Peinado C., Salvador E.F., Corrales T., Bosch P., Catalina F. (2004). Fluorescent probes for monitoring the pulsed-laser-induced photocuring of poly(urethane acrylate)-based adhesives: Fluorescent probes. J. Polym. Sci. Part A Polym. Chem..

[56] Scherzer T., Decker U. (1999). Real-time FTIR–ATR spectroscopy to study the kinetics of ultrafast photopolymerization reactions induced by monochromatic UV light. Vib. Spectrosc..

[57] Paczkowski J., Neckers D.C. (1993). New fluorescence probes for monitoring the kinetics of laser-initiated polymerization. J. Polym. Sci. Part A Polym. Chem..

[58] Liu M., Li M.-D., Xue J., Phillips D.L. (2014). Time-resolved spectroscopic and density functional theory study of the photochemistry of irgacure-2959 in an aqueous solution. J. Phys. Chem. A.

[59] Kim S.W., Bae Y.H., Okano T. (1992). Hydrogels: Swelling, drug loading, and release. Pharm. Res..

[60] Rosu D., Rosu L., Cascaval C.N. (2009). IR-change and yellowing of polyurethane as a result of UV irradiation. Polym. Degrad. Stab..

[61] Marchioli G., Zellner L., Oliveira C., Engelse M., de Koning E., Mano J., Karperien, van Apeldoorn A., Moroni L. (2017). Layered PEGDA hydrogel for islet of langerhans encapsulation and improvement of vascularization. J. Mater. Sci. Mater. Med..

[62] Liu S., Yeo D.C., Wiraja C., Tey H.L., Mrksich M., Xu C. (2017). Peptide delivery with poly(ethylene glycol) diacrylate microneedles through swelling effect. Bioeng. Transl. Med..

[63] Allen G. (1996). Comprehensive Polymer Science and Supplements.

[64] Berlman I. (1965). Handbook of Fluorescence Spectra of Aromatic Molecules.

[65] Ware W.R. (1962). Oxygen quenching of fluorescence in solution: An experimental study of the diffusion process. J. Phys. Chem..

[66] Hintermann L. (2010). Comprehensive organic name reactions and reagents. Angew. Chem. Int. Ed..

[67] Andrzejewska E. (2001). Photopolymerization kinetics of multifunctional monomers. Prog. Polym. Sci..

[68] Akram N., Mansha A., Premkumar R., Benial A.M.F., Usman M., Rasool N., Asim S. (2020). Spectroscopic, quantum chemical and molecular docking studies of 2-Hydroxy-4′-(2-hydroxyethoxy)-2-methylpropiophenone: A potent anti-Alzheimer’s drug. Chem. Data Collect..

[69] Ma Z., Niu X., Xu Z., Guo J. (2014). Synthesis of novel macrophotoinitiator for the photopolymerization of acrylate. J. Appl. Polym. Sci..

[70] Atkins P.W., De Paula J. (2009). Elements of Physical Chemistry.

[71] Schneider C.A., Rasband W.S., Eliceiri K.W. (2012). NIH Image to ImageJ: 25 years of image analysis. Nat. Methods.

[72] Technical Committee (2009). ISO/TC 194 part 5: Tests for in vitro cytotoxicity. ISO 10993-5:2009 Biological Evaluation of Medical Devices.

